# An Emerging Role for Neutrophil Extracellular Traps in IgA Vasculitis: A Mini-Review

**DOI:** 10.3389/fimmu.2022.912929

**Published:** 2022-06-21

**Authors:** Xiu-Qi Chen, Li Tu, Qing Tang, Li Huang, Yuan-Han Qin

**Affiliations:** Department of Pediatrics, The First Affiliated Hospital, Guangxi Medical University, Nanning, China

**Keywords:** IgA vasculitis, neutrophil extracellular traps, pathogenesis, biomarker, neutrophils, IgA vasculitis nephritis

## Abstract

Immunoglobulin A vasculitis (IgAV) is the most common systemic small vessel vasculitis in childhood. Its clinical manifestations are non-thrombocytopenic purpura, accompanied by gastrointestinal tract, joint, kidney and other organ system involvement. The pathogenesis of IgAV has not been fully elucidated. It may be related to many factors including genetics, infection, environmental factors, and drugs. The most commonly accepted view is that galactose-deficient IgA1 and the deposition of IgA and complement C3 in small blood vessel walls are key contributors to the IgAV pathogenesis. Extensive neutrophil extracellular traps (NETs) in the peripheral circulation and skin, kidney, and gastrointestinal tissue of patients with IgAV has been identified in the past two years and is associated with disease activity. This mini-review provides a possible mechanism for NETs involvement in the pathogenesis of IgAV.

## Introduction

Immunoglobulin A vasculitis (IgAV), also known as Henoch‐Schönlein purpura, is an inflammatory small vascular disease involving the capillaries, venules, or arterioles (1). The clinical manifestations of IgAV are non-thrombocytopenic purpura, mainly involving the skin, gastrointestinal tract, joint and kidneys, and deposition of IgA or IgA-immune complexes (IgA-ICs) in the vascular wall. The incidence of IgAV in children is 10-27/100,000 per year (2). The majority of cases occur between 2 and 10 years of age, with a peak onset between 4 and 7 years of age (3, 4). In most children, IgAV is self-limited and has a good prognosis, but a few cases have renal involvement and a recurrent or even prolonged course. IgAV nephritis (IgAVN), which is a major cause of mortality, is the cause of 1%–2% of pediatric end-stage renal disease cases (3). The pathogenesis of IgAV has not been fully elucidated. At present, it is believed to be caused by genetics, infection, environmental factors, drugs, and other factors (5). There is a wide range of immunological abnormalities in IgAV.

## The Physiological Function of NETs and Their Role in Autoimmune Diseases

Neutrophils are the most abundant white blood cells in the human peripheral circulation. They play a key role in the innate immune system and constitute the body’s first line of defense against pathogens. Previous studies have shown that neutrophils phagocytose and kill bacteria directly through secretion of proteolytic enzymes, antibacterial proteins and reactive oxygen species (ROS), which are directly to kill bacteria (6, 7).A novel mechanism of neutrophil defense against infection through release of neutrophil extracellular traps (NETs) was reported in recent years (8). This process fundamentally differs from both cell death and necrotizing apoptosis and is called NETosis (9). At present, there are two mechanisms by which NETs are formed: suicide lytic NETosis and vital NETosis. In lytic NETosis, neutrophils release NETs through cell membrane lysis death, which depends on the Raf/MEK/ERK signaling pathway and the activation of NADPH oxidase. In lyticNETosis, the cell membrane breaks down and neutrophils are unable to secrete particles. In vital NETosis, DNA from the nucleus erupts in vesicles, passes through the cytoplasm and binds to the plasma membrane, transporting DNA outside the cell to formation of NETs without damaging the membrane and maintaining the integrity of neutrophil (10). The structure of NETs directly wraps around invading microorganisms and uses its highly concentrated antimicrobial peptides to degrade virulence factors and kill pathogenic microorganisms, preventing the spread and dissemination of infection, which plays an important role in infection defense (8). However, excessive formation of NETs and clearance of obstacles also has a toxic effect on the host. NETs related components, such as nucleic acids and proteins, were exposed as autoantigens in the inflammatory environment, which can stimulate the autoimmune response of susceptible individuals and promote various autoimmune diseases (11).

## NETs Involved in the Pathogenesis of IgAV

In 2020 Bergqvist, C et al. (12) reported that NETs were significantly increase in skin tissues in the early stages of IC-mediated small vasculitis, such as allergic vasculitis and IgAV. Our previous study evaluated the level of NETs in the peripheral blood and gastrointestinal and renal tissues of children with IgAV at different periods. The study evaluated components of NETs, which included cell-free DNA (cf-DNA), myeloperoxidase-DNA (MPO-DNA), citrullinated-histone H3 (cit-H3), neutrophil elastase (NE), and cathelicidin antimicrobial peptides (CAMP, LL37). The level of NETs significantly increased in children with IgAV onset and active stage, while the level of NETs gradually returned to normal in children in the remission stage and drug withdrawal (13). In autoimmune diseases, excessive NETs are known to act as an exposed autoantigen *in vivo*, inducing the production of autoantibodies, thereby increasing the intensity of the inflammatory response. A continuous increase in NETs indicates a high inflammatory state, and reflects the imbalance between the formation and clearance of NETs in IgAV, leading to the accumulation of excessive NETs, which ultimately leads to autoimmune disorders, chronic inflammation and tissue damage. These processes have been associated with the development of autoimmune and inflammatory diseases (14). Several studies have shown that NETs are involved in the development and progression of autoimmune diseases such as ANCA-associated vasculitis, rheumatoid arthritis (RA), inflammatory bowel disease and systemic lupus erythematosus (SLE) (15–18). A recent study reported that MPO-DNA is significantly elevated in the circulation of patients with IgAV and positively correlates with IgA levels, which suggests that NETs are involved in the pathogenesis of IgAV (19). NETs may influence the activity or severity of IgAV (13, 19).

## Mechanism of NETs in IgAV

### Disordered Equilibrium Between NETs and DNase I

Our previous study (13) revealed that serum degradation of NETs significantly declines in children with IgAV onset and active IgAV. Children in drug withdrawal had a normal level of NETs degradation. The level of DNase I also decreases in children with IgAV onset and active IgAV. The reduced ability to degrade NETs is negatively correlated with the presence of DNase I, which is required to degrade NETs (20). The decreased activity of DNase I may be one of the reasons for the significant increase in NETs and thus may cause immune imbalance (21). In patients with SLE and eosinophilic granuloma, the ability of the extracellular and intracellular environment to degrade DNA is significantly reduced. This phenomenon seems to be a common characteristic of autoimmune diseases (22, 23). In addition, over-activation of complement system and over deposition of complement protein C1q also inhibit the production of DNase I, resulting in ineffective NETs degradation (24). Therefore, excessive NETs formation is related to deficient DNase I activity, which leads to disorders that promote immunological homeostasis dysregulation and tissue damage (25). Impaired self-degradation of NETs is associated with RA and lupus nephritis (26–29).The decreased activity of DNase I eventually leads to a reduced ability to degrade NETs, which is one of the reasons for the increase in NETs in IgAV.

### Aberrant Glycosylation of IgA1 and IgA-ICs Induces NETs Formation in IgAV

Deposition of IgA on the vascular wall is characteristic of IgAV. IgA activate neutrophils and release NETs into tissues and the peripheral blood. Studies have shown that NETs are involved in various IC-mediated small vasculitis conditions, and that circulating NETs are related to the severity of vascular inflammation (12). In renal biopsies from patients with ANCA-associated vasculitis, the formation of NETs was found in the involved glomeruli and stroma lesions (30). IgA can induce neutrophils to release NETs *via* Fcα receptor I (FcaRI) (31). FcaRI is elevated in children with active IgAV (32). In idiopathic IgA nephropathy, proteinuria and leukocyte infiltration are more pronounced, and FcaRI activation leads to a more severe inflammatory response. It is believed that FcaRI promotes and aggravates tissue and kidney damage by activating the cascade reaction of cytokines and chemokines (33). NETs have been shown to induce an autoimmune response in other autoimmune diseases such as SLE and ANCA-associated vasculitis (34–36). In addition, in patients with RA, the level of circulating NETs is positively correlated with the severity of periodontitis (37).

**Figure 1:**
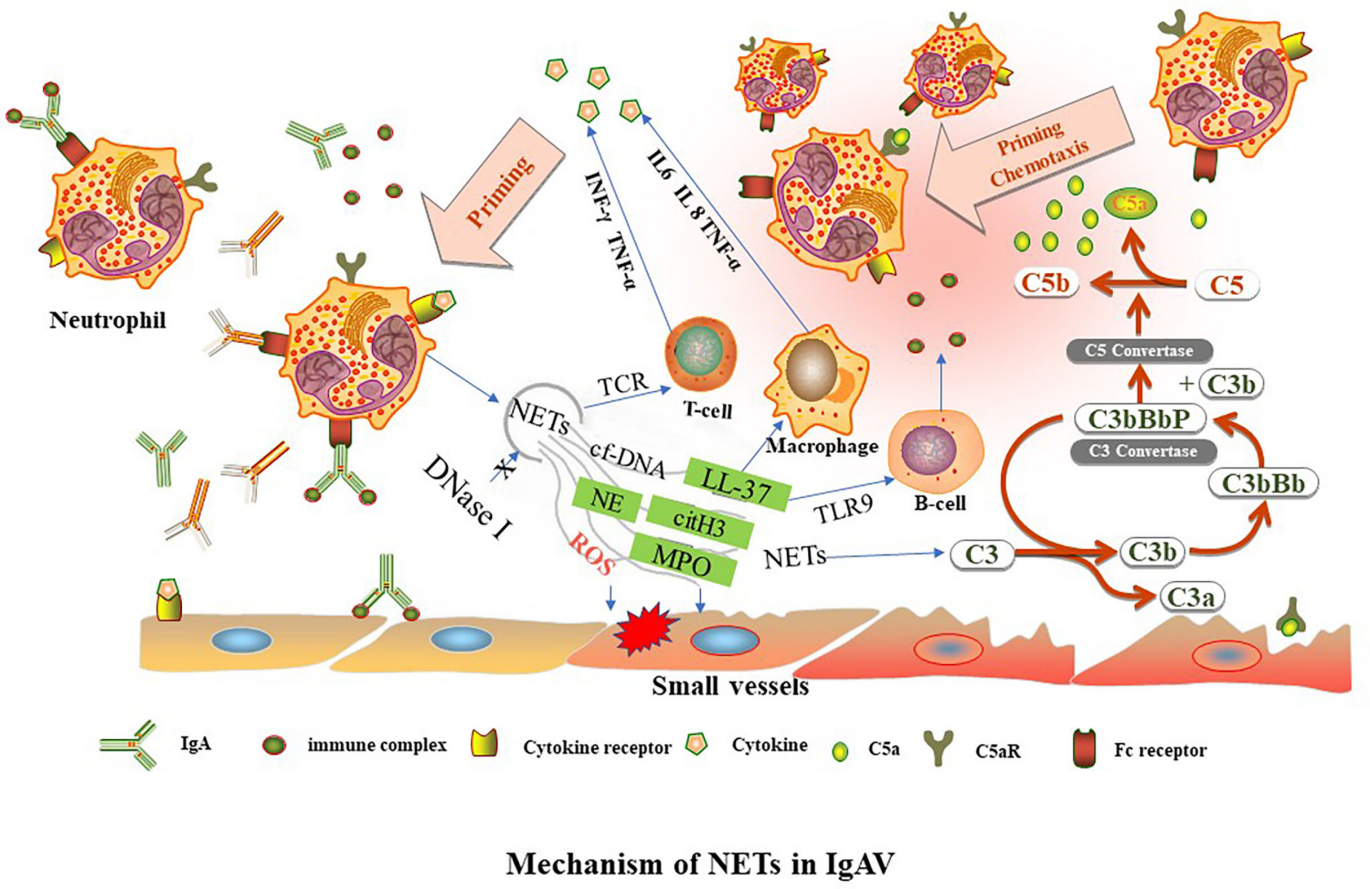
TNFα, tumor necrosis factor alpha. INF-γ, interferon gamma. NETs, neutrophil extracellular traps. LL37, cathelicidin antimicrobial peptides. C3, complement factor 3. IL, interleukin, MPO, myeloperoxidase. NE, neutrophil elastase. cit-H3, citrullinated-histone H3. cf-DNA, cell free DNA. TCR, T cell receptor. TLR9, toll-like receptor 9-dependent manner. Aberrant glycosylation of IgA1 and IgA immune complexes (IgA-ICs) induce NETs formation by binding to the Fc receptor of neutrophils. The level of DNase I decreases, leading to reduce of NETs degradation. NETs activate downstream target immune cells to release cytokines. NETs can activate immune-related cells, such as T lymphocytes (through TCR), B lymphocytes (through TLR9-dependent manner) and macrophages to release cytokines, such as IL6, IL 8, TNF-α, and INF-γ. NETs are involved in different complement bypass pathways.

NETs formation has been detected in tissue biopsies of patients in the early stages of IC-mediated vasculitis (14). These immobilized ICs induce human neutrophils to release NETs *in vitro* (38). The formation of NETs increases in the renal, gastric, and duodenal tissues of children during IgAV onset and active IgAV, which may be related to IgA-ICs deposition activating neutrophils to release NETs. In lupus nephritis, deposition of circulating ICs in the glomerular basement membrane is accompanied by the accumulation of NETs in the tissue, resulting in tissue damage (39). The deposition of IgA and C3 and the formation of NETs are common in the renal, gastric and duodenal tissues of children with IgAV. It is speculated that IgA-ICs and C3 deposition may be involved in the occurrence and development of IgAV through various mechanisms, such as complement activation, chemotaxis infiltration and aggregation of neutrophils to promote the release of NETs (**Figure 1**).

### NETs Activate Downstream Target Immune Cells to Release Cytokines

Neutrophils release related components of NETs such as MPO and protease, which further aggravates tissue damage. Similarly, IgA binds to the FcαRI junctions of neutrophils, releasing tumor necrosis factor α (TNF-α), leukotriene B4 (LTB4), etc. (40, 41). TNF-α can further stimulate endothelial cells to produce interleukin (IL)-8 (42). IgA may activate neutrophils to release IL-8 (1, 43). Studies show that LTB4 is significantly increased in children with IgAV (44). LTB4 can induce further neutrophil migration through a positive feedback pathway (40). Although LTB4 has no direct effect on microvascular injury of inflammatory tissues, it can make white blood cells adhere to vascular endothelial cells, resulting in increased vascular permeability and aggravating tissue injury (45). LTB4 plays an important role in inflammation, the immune system, and allergies. TNF-α is a pro-inflammatory factor involved in the occurrence and development of IgAV and is closely related to kidney damage. TNF-α can even reflect the degree of renal damage in IgAVN (46, 47). In addition, NETs-related components can activate immune-related cells such as B lymphocytes, T lymphocytes and antigen-presenting cells to release IL-6, IL-8, interferon γ (INF-γ), and TNF-α (14). In the interleukin family, IL-6, IL-8, IL-10, and IL-33 are all related to IgAV (46, 48–50). IL-2 is negatively correlated with the severity of the disease (51). IL-6 promotes the activation of B cells and the production of relevant antibodies, which are mainly deposited in the mesangial region of the kidney. Through the action of T cells, IL-6 stimulates the proliferation and fibrosis of mesangial tissues, aggravates kidney damage and leads to the occurrence and development of IgAVN (52). IL-10 plays a protective role by inhibiting the antigen presentation function of macrophages and indirectly inhibiting the function of natural killer cell (53). Under the stimulation of IL-8, an increase in the cytoplasmic Ca^2+^ of neutrophils mediates the release of hydrogen peroxide in a respiratory burst reaction, and lysosomal enzymes can be released through chemotaxis of neutrophils, leading to capillary destruction (46). IgA can activate the complement system through bypass and lectin pathways. The levels of C3a and C5a increase in the circulation of IgAV, and C3 and C5-C9 are deposited in the skin tissue and mesangial region of glomeruli (54, 55). These compounds can form membrane-attacking complexes that directly destroy the membranes of target cells, and deposition of C4d and C5B-9 in the kidney is associated with poor prognosis (56). NETs activate C3 and eventually convert it to C5a, which can induce chemotaxis and neutrophil aggregation, and stimulate endothelial cells to secrete IL8.

## NETs May Be a Potential Biomarker to Assess Disease Activity in IgAV

NETs have been reported as a marker of disease activity in other diseases. The One predictor of inflammatory response and sepsis is cf-DNA (57, 58). Cit-H3 is a useful biomarker for early detection of liver dysfunction (59). NETs can be used as markers and therapeutic targets for ophthalmic diseases including dry eye, glaucoma, age-related macular degeneration, and diabetic retinopathy (60, 61). NETs and anti-NETs associated antibodies are indicators of SLE activity (11, 16). NETs significantly increase during IgAV onset and the active stage of IgAV but decrease in the remission and withdrawal stage of IgAV (13). Most patients in the active and relapse have IgAVN. It is speculated that changes in NETs levels may reflect disease activity of IgAV in children, especially those with IgAVN need corticosteroids or immune suppressive therapy.

There are no widely used biomarkers to predict disease activity or the prognosis of IgAVN. The combined indexes of blood examination, immunoglobulin, C-reactive protein, procalcitonin and trace elements have been used to predict the index (62–64). The detection of related metabolites in urine has also been considered. The soluble transferrin receptor concentration in urine increases significantly during the active stage of IgAVN, but the correlation coefficient is low (65). The ratio of urine (Fcα receptor × glutamine transferase)/urine protein and perforin 3 has also been used to predict disease activity, but clinical detection methods for these markers are limited (32, 66). The severity of IgAVN is correlated with alpha-smooth muscle actin (α-SMA) and C-Met, while IgAV with gastrointestinal involvement is correlated with fecal calprotectin, D-dimer and fibrin degradation products (67–69). Therefore, NETs may be a potential convenient biomarker and indicator of IgAVN disease activity, particularly in those patients who would have an ominous outcome.

NETs related components include cf-DNA, MPO-DNA, and NE. Peripheral blood cf-DNA is simple and convenient to measure, but Moss et al. (70) showed that cf-DNA can be released from a variety of cells other than neutrophils during inflammation. Whether cf-DNA alone can predict the level of NETs needs more research. Therefore, the use of NETs or their related components as biomarkers for disease still needs further study for confirmation.

## Conclusion and Perspectives

In conclusion, IgA or IgA-ICs can activate neutrophils to release NETs. NETs-related components can directly damage tissues or secrete large amounts of cytokines by activating downstream target immune cells. Cytokines can aggravate tissue damage and cause neutrophil aggregation, forming a vicious cycle (**Figure 1**). NETs may be a potential biological indicator to assess disease activity in children with IgAV.

However, many unanswered questions about the mechanism of NETs in IgAV remain. The mechanism by which neutrophils mediate IgAV tissue damage is not completely clear. At present, the specific mechanism for NETs signaling pathway regulation and NETs related components in IgAV-induced tissue injury has not been elucidated. Which signaling pathway IgA/FcαR regulates the formation of NETs in neutrophil remains unclear.

## Data Availability Statement

The raw data supporting the conclusions of this article will be made available by the authors.

## Author Contributions

XQC were responsible for the conception, design and drafted the manuscript. QT and LH were responsible for design of the review. LT and YHQ revised the manuscript. All authors contributed to the article and approved the submitted version.

## Funding

This study was supported by the Innovation Project of Guangxi Graduate Education (no. YCBZ2021047), the Research Basic Ability Enhancement Project for Young and Middle-aged Teachers in Guangxi Universities (no: 2021KY0096).

## Conflict of Interest

The authors declare that the research was conducted in the absence of any commercial or financial relationships that could be construed as a potential conflict of interest.

## Publisher’s Note

All claims expressed in this article are solely those of the authors and do not necessarily represent those of their affiliated organizations, or those of the publisher, the editors and the reviewers. Any product that may be evaluated in this article, or claim that may be made by its manufacturer, is not guaranteed or endorsed by the publisher.
